# Supplementary data on virus-like particles in the brainstem of Parkinson’s disease patients and controls

**DOI:** 10.12688/f1000research.20504.2

**Published:** 2022-01-17

**Authors:** Robert R. Dourmashkin, Peter Locker, Sherman A. McCall, Matthew J. Hannah

**Affiliations:** 1 Virus Reference Dept., National Infection Service, London, London, NW9 5EQ, UK; 2 London Metropolitan University, Tower Hamlets, London, E1 7QA, UK; 3 Deprtment of Molecular Pathology, Armed Forces Institute of Pathology, Washington DC, USA; 4 Electron Microscopic Service, Virus Reference Dep’t. National Infection Service, London, UK

**Keywords:** Virus-like particles, Parkinson’s disease, transmission electron microscope imaging

## Abstract

In this study, we present 77 transmission electron microscopy (TEM) images of human brainstem tissue from 11 cases of late onset Parkinson’s disease (PD). The tissues were fixed, embedded, sectioned, and stained for TEM application. In addition, we present 11 images from autopsy specimens of 1 case of human poliomyelitis infection as positive controls and 12 images from 8 cases of autopsy specimens of other conditions as negative controls. In the TEM images of the PD cases there were cytoplasmic inclusion bodies consisting of virus-like particles (VLP) 30 nm in diameter that were associated with endoplasmic reticulum membranes.  In the nuclei of the PD neurons there were VLP ranging from 40 nm to 50 nm in diameter. In the poliomyelitis cases, similar particles as were observed in PD which were interpreted to be poliomyelitis virus particles. In the negative controls one case was identified which showed similar VLP (Figure 1, controls).  A Lewy body was found in this “control” case (Figure 10) suggesting that this was an undiagnosed case of PD. Cytoplasmic ribosomes measuring approximately 17 nm were observed in the control neurons.

## Introduction

In our previous communication, we presented a number of TEM images selected from our work on PD. We also presented our immunohistochemical results in which we detected enterovirus antigen in PD brainstem tissue ^1^. In order to assist in the interpretation of our TEM results, we now present a larger number of TEM images from PD archives together with relevant controls. We also include TEM images of Lewy bodies from the cases of PD, the diagnostic sign of PD (see *Underlying data*) ^2^.

## Methods

The methods for TEM were the same as those employed in our previous communication ^1^. Included were 8 control cases, 1 case of poliomyelitis and 11 cases of Parkinson’s disease. The tissue samples from the control cases were obtained from the John Radcliffe Hospital, Department of Neuropathology. The tissue samples from the PD cases and from the poliomyelitis case were from the Armed Forces Institute of Pathology, in Washington DC, now closed.

### Demographic information controls

Controls: Case 06/126: 87 y. o. female, no cause of death known; Case 06/110: 70 y. o. male, prostate cancer; Case 06/112: 87 y. o. male, perforated gastric ulcer; Case 05/66: 26 y. o. female, cystic fibrosis; Case 05/152: 51 y. o. female, stomach cancer. All PD cases died of PD. The polio case was a 32 y. o. male that died of poliomyelitis.

### Ethical clearance

Study of brain specimens had been cleared for ethical agreement by the National Ethics Committee for Oxford UK, rec. no. 07/H0606/85. The brain samples from human autopsy material were obtained from the pathology collections of the John Radcliffe Hospital, Oxford UK and the former Armed Forces Institute of Pathology, Washington DC USA. These institutions approved the use of the tissues for research and they were satisfied that no further ethical approval was required. In the case of the material from the UK, the principal author releases the “tissue disclaimer” from the Thomas Willis Oxford collection, at the Neuropathology Department.

## Conclusions

We note the role α-synuclein plays in the pathogenesis of PD ^3^. It is conceivable that a virus is involved with α-synuclein in the pathogenesis of PD. We suggest that virologists undertake the isolation of infectious virus from diseased PD.

Supplementary data on virus-like particles in the brainstem of Parkinson’s disease patients and controls In this study, we present 77 transmission electron microscopy (TEM) images of human brainstem tissue from 11 cases of late onset Parkinson’s disease (PD). The tissues were fixed, embedded, sectioned, and stained for TEM application. In addition, we present 11 images from autopsy specimens of 1 case of human poliomyelitis infection as positive controls and 12 images from 8 cases of autopsy specimens of other conditions as negative controls. Click here for additional data file. Copyright: © 2022 Dourmashkin RR et al. 2022

## Data availability

### Underlying data

Figshare: Supplementary data on virus-like particles in the brainstem of Parkinson’s disease patients and controls. https://doi.org/10.6084/m9.figshare.18618059.v1 ^2^.

This project contains the following underlying data:

Legends for TEM images of PD brain.

- Parkinson’s Figure 1. Low magnification of a PD neuron, original image 2.13b015. Cytoplasmic inclusion bodies are shown (arrows).
- Parkinson’s Figure 2. Low magnification of a PD neuron of the same case, image 2.13b025. Cytoplasmic inclusion bodies are shown (arrows).
- Parkinson’s Figure 3. High magnification of a cytoplasmic neuron of the same case, image 2.13b028. Multiple VLP are shown. (arrows)
- Parkinson’s Figure 4. Low magnification of a PD neuron of the same case, image 2.13b032 Cytoplasmic inclusion bodies (arrows) and external inclusion bodies are shown.
- Parkinson’s Figure 5. High magnification of a cytoplasmic inclusion body in a PD neuron in the same case, image 2.13b031. Many VLP are shown. (arrows)
- Parkinson’s Figure 6. High magnification of a cytoplasmic inclusion body in a PD neuron in the same case, image 2. 13b033. Many VLP are shown.
- Parkinson’s Figure 7. High magnification of a cytoplasmic inclusion body in a PD neuron in the same case as above, image 2.13b046. There are proliferating cytoplasmic membranes and VLP associated with membranes. (arrows)
- Parkinson’s Figure 8. Low magnfication of a PD neuron in the same case. Inclusion body is shown (arrows). Image 2.13b055.
- Parkinson’s Figure 9. High magnification of an inclusion body in the PD neuron in the same case, image 2.13b060. Many VLP are shown.
- Parkinson’s Figure 10. Low magnification of a neuron in the same case, image 2.13b072. Cytoplasmic inclusion bodies are shown (arrows).
- Parkinson’s Figure 11. Image of a Lewy body in the same case, image 2.13c012.
- Parkinson’s Figure 12. Low magnification of a PD neuron in the same case, image 2.13013. Cytoplasmic inclusion body is indicated (arrows).
- Parkinson’s Figure 13. Image of a cytoplasmic inclusion body showing proliferation of cytoplasmic membranes and VLP. (arrows). Image 2.13c017.
- Parkinson’s Figure 14. Measurements of intranuclear VLP in a PD neuron. NM=nuclear membrane. Image 2.13004.
- Parkinson’s Figure 15. Low magnification of a PD neuron. Cytoplasmic inclusion bodies are indicated (arrows). Image 2.13028.
- Parkinson’s Figure 16. In another PD case, low magnification image of damaged neuroglia. Image 3.08010.
- Parkinson’s Figure 17. In the same PD case, a Lewy body. Image 03.08049.
- Parkinson’s Figure 18. At higher magnification, an image of the same inclusion body shown in fig.26, consisting of VLP. Image 3.02002
- Parkinson’s Figure 19. In the same PD case, an image of a neuron with multiple VLP close to the internal face of the nuclear membrane (arrows). Image 3.13007.
- Parkinson’s Figure 20. In the same PD case, two VLP are shown budding from the internal face of a nucleus (arrows.) Image 3.13016.
- Parkinson’s Figure 21. In the same PD case, a high magnification image shows multiple VLP arranged on an endoplasmic membrane in a cytoplasmic inclusion body. Image 3i.13.09006.
- Parkinson’s Figure 22. In the same PD case, measurements of intranuclear VLP. Image 3i.13.09021.
- Parkinson’s Figure 23. In another PD case, at high magnification, an image of multiple VLP in a cytoplasmic inclusion body. Image 4.08b003.
- Parkinson’s Figure 24. In the same PD case as above, an image of VLP replicating on endoplasmic membranes. N = nucleus, C = cytoplasm. Image 4. 08b006.
- Parkinson’s Figure 25. In the same case, a low magnification image of a PD neuron showing cytoplasmic inclusion bodies (arrows). Image 4.08b009.
- Parkinson’s Figure 26. In the same case, a low magnification image of a PD neuron showing cytoplasmic inclusion bodies (arrows). Image 4.08b014
- Parkinson’s Figure 27. In the same case, a high magnification image, measurements of intranuclear VLP. Image 4.08b016.
- Parkinson’s Figure 28. In the same case, intranuclear VLP are shown (arrows). Image 4.08b021.
- Parkinson’s Figure 29. In the same case, intranuclear VLP are shown (arrows). Image 4.08b022.
- Parkinson’s Figure 30. In the same case, intranuclear VLP are shown (arrows). Image 4.08b030.
- Parkinson’s Figure 31. Measurements are shown. Image 4.08b032.
- Parkinson’s Figure 32. In the same case, a low magnification image of the same PD case shows multiple cytoplasmic inclusion bodies. Image 4.08b036.(arrows)
- Parkinson’s Figure 33. In another PD case, a high magnification image of intranuclear VLP emerging from virus matrix. (arrows). Image 4.08001.
- Parkinson’s Figure 34. Another image of the same area in the proximity of the nucleolus. Image 4.08003
- Parkinson’s Figure 35. Another image of the same area as Fig. 34. Image 4.08005.
- Parkinson’s Figure 36. Another PD case. Intranuclear VLP situated close to the nuclear membrane (arrow). Image 4.08009.
- Parkinson’s Figure 37. Another PD case. A cytoplasmic inclusion body. Image 4.08015.(Arrow)
- Parkinson’s Figure 38. The same PD case as Fig. 43. Intranuclear VLP (arrows). Image 4.08017.
- Parkinson’s Figure 39. Parkinson’s disease cases, neurons of the basal ganglia. Image of a Lewy body. Image 5b.0801.0
- Parkinson’s Figure 40. Clusters of VLP in a cytoplasmic inclusion body. (arrows). Image 5D.08002.
- Parkinson’s Figure 41. Large intranuclear VLP. Image 5D.08004.
- Parkinson’s Figure 42. Same image, with measurements. Image 5D.08005.
- Parkinson’s Figure 43. Same PD case. Large intranuclear VLP close to and adhering to the internal face of the nuclear membrane. (arrows). Image 5D.08006.
- Parkinson’s Figure 44. Same PD case. Similar VLP adhering to the internal face of the nuclear membrane. (arrows). Image 5D08007.
- Parkinson’s Figure 45. Same PD case. Measurements of VLP in a cytoplasmic inclusion body. Image 5D.08018.
- Parkinson’s Figure 46. Same PD case. Measurements of VLP in a cytoplasmic inclusion body. Image 5D.08028.
- Parkinson’s Figure 47. Same PD case. Arrows indicating membranes in a cytoplasmic inclusion body. Image 5D08017.
- Parkinson’s Figure 48. Another PD case. Intranuclear polyribosomes. Image 6.08002.
- Parkinson’s Figure 49. Higher magnification of similar image, with measurements. Image 6.08004.
- Parkinson’s Figure 50. In the same PD case, a Lewy body. Image 6.08011.
- Parkinson’s Figure 51. High magnification of a Lewy body. Image 6.08013.
- Parkinson’s Figure 52. Low power of neuron from another PD case, with cytoplasmic inclusion bodies (arrows). Image 7.08b001.
- Parkinson’s Figure 53. Low magnification of a cell from the same PD case with a Lewy body Image 7.08019.
- Parkinson’s Figure 54. Low magnification of neuron from another PD case, with cytoplasmic inclusion bodies (arrows). Image 8.08001.
- Parkinson’s Figure 55. Same case, high magnification of neuron with large intranuclear VLP (arrows). Image 8.08008. N=Nucleus.
- Parkinson’s Figure 56. Same area of nucleus, (arrows). Image 8.08009.
- Parkinson’s Figure 57. Same PD case, large intranuclear VLP. Image 8.08025.
- Parkinson’s Figure 58. Same PD case, low magnification of a neuron with cytoplasmic inclusion bodies (arrows). Image 8.08034.
- Parkinson’s Figure 59. Same PD case, budding intranuclear VLP. Image 8.08036.
- Parkinson’s Figure 60. Same PD case, intranuclear VLP budding from the internal leaf of the nuclear membrane. Measurements, 8.08040.
- Parkinson’s Figure 61. Same PD case, intranuclear VLP. Measurements, image 8.08049.
- Parkinson’s Figure 62. Another PD case. Cytoplasmic VLP. Image 9.08016.
- Parkinson’s Figure 63. A PD case. Intranuclear VLP budding from internal nuclear membrane of neuron. Image 10.08a001.
- Parkinson’s Figure 64. Same PD neuron, measurements. Image 10.08a005.
- Parkinson’s Figure 65. Same PD endothelial cell. High magnification showing large numbers of clustered small VLP. Image 10.08a008.
- Parkinson’s Figure 66. Same PD endothelial cell, high magnification. Large numbers o0f small VLP. Image 10.08a019.
- Parkinson’s Figure 67. A PD case, showing large VLP in the cytoplasm and measurements. Image 13.09c006.
- Parkinson’s Figure 68. A PD case. A Lewy body is shown in the cytoplasm of s neuron. Image 13.09c010.
- Parkinson’s Figure 69. A PD case. A Lewy body free in the neuropil. Image 13.09019.
- Parkinson’s Figure 70. A PD case. Large VLP in the nucleus and cytoplasm of a neuron. Image 13a09009. (arrows).
- Parkinson’s Figure 71. Another PD case. A Lewy body free in the neuropil. Image 14.09b006.
- Parkinson’s Figure 72. Same PD case. Small intranuclear VLP clustered in groups. Image 14.09c035. (N=nucleolus).
- Parkinson’s Figure 73. Same PD case. Small intranuclear VLP, clustered in groups. Image 14.09c045.
- Parkinson’s Figure 74. Same PD case. Large intranuclear VLP budding from membranes. (arrows). Image 14.431037.
- Parkinson’s Figure 75. Another PD case. Large intranclear VLP (arrow). Image 16.09c001.
- Parkinson’s Figure 76. Higher magnification of same area. Image 16.09c005.
- Parkinson’s Figure 77. In the same PD case, large intranuclear VLP. (arrows). Image 16.09c014.

Legends of TEM images of poliomyelitis spinal cord.

- Poliomyelitis Figure 1. A case of human poliomyelitis. Large numbers of small virus particles embedded in a matrix, at the periphery of a neuron. (arrows). Image 5a.13021.
- Poliomyelitis Figure 2. Same polio case. Clusters of small virus particles in an inclusion body. (arrows). Image 5a.13022.
- Poliomyelitis Figure 3. Same polio case, low magnification. Neuron in apoptosis. Virus inclusion body (arrow). Image 5a.13025.
- Poliomyelitis Figure 4. Same polio case. Intranuclear virus particles (arrow). Image 5a.13026.
- Poliomyelitis Figure 5. Same image as Fig. 4, measurements of virus particles. Image 5a.13027.
- Poliomyelitis Figure 6. High magnification of a polio inclusion body, showing clusters of virus particles, both free and associated with endoplasmic membranes. (arrows). Image 5a.13029.
- Poliomyelitis Figure 7. Case of human poliomyelitis. Clusters of virus particles, embedded in amorphous matrix. (arrows). Image 48.05006.
- Poliomyelitis Figure 8. Case of human poliomyelitis. Cytoplasmic virus particles. Image II.48.05001.
- Poliomyelitis Figure 9. Case of human poliomyelitis. Image II.48.05002 Low magnification. (arrow)
- Poliomyelitis Figure 10. Case of human poliomyelitis. Low magnification. Image II48.05003. Inclusion body shown. (arrow)
- Poliomyelitis Figure 11. Case of human poliomyelitis. Virus particles embedded in amorphous matrix, associated with cytoplasmic membranes. Image II.48.05005.

Legends of TEM images of control brain.

- Control Figure 1. Ribosomes in Nissl body, and mitochondrion. Measurements, Image 9.090117.
- Control Figure 2. Control case. Ribosomes in a Nissl body. Measurements, image 9.08039.
- Control Figure 3. Control case. Nissl body. Image 10.09b004.
- Control Figure 4. Control case; a group of ribosomes in a neuron in the basal ganglia. Measurements, image 10.09015.
- Control Figure 5. Control case; a group of ribosomes in a Nissl body in the same area as above. Image 10a09009.
- Control Figure 6. Measurements. Control case, as above; endoplasmic reticulum in the cytoplasm of a neuron. Image 10c.09011.
- Control Figure 7. A case that was reported as being a control case. VLP characteristic of PD are shown (arrows). Image 11.09b001.
- Control Figure 8. In a same case as in Figure 9, an intracellular Lewy body. Image 11.09006.
- Control Figure 9. A control case, showing cytoplasm and nucleus. Image 12.09015.
- Control Figure 10. Control case. Image 1209b004.
- Control Figure 11. Control case. Image 1209b015.
- Control Figure 12. Control ribosomes, measurements. Image x10.09011.

Data are available under the terms of the Creative Commons Attribution 4.0 International license (CC-BY 4.0).
