# Supplementary material for: Supplementary data on virus-like particles in the brainstem of Parkinson’s disease patients and controls
Source: F1000Res. 2022 Jan 17;9:692. Originally published 2020 Jul 9. [Version 2] doi: 10.12688/f1000research.20504.2 (PMC8958977; doi:10.12688/f1000research.20504.2)
Supplement: Peer review history

# Peer review: Reviewer response for version 2

**DOI:** 10.5256/f1000research.79367.r120080

**Published:** 2022-03-17

**Authors:** Marie-Eve Tremblay

**Affiliations:** 1 Division of Medical Sciences, University of Victoria, Victoria, British Columbia, Canada

Thank you for the revision! All concerns have been addressed and I am pleased to give this version final approval.

Are sufficient details of methods and materials provided to allow replication by others?

Yes

Is the rationale for creating the dataset(s) clearly described?

Partly

Are the datasets clearly presented in a useable and accessible format?

No

Are the protocols appropriate and is the work technically sound?

Partly

Reviewer Expertise:

brain ultrastructure, microglia, neurons, synapses, animal models, human post-mortem samples, stress, aging, neurodegenerative diseases

I confirm that I have read this submission and believe that I have an appropriate level of expertise to confirm that it is of an acceptable scientific standard.

# Peer review: Reviewer response for version 2

**DOI:** 10.5256/f1000research.79367.r120081

**Published:** 2022-01-19

**Authors:** Doris Bucher

**Affiliations:** 1 Department of Microbiology and Immunology, New York Medical College, Valhalla, NY, USA

All concerns have been addressed and I am pleased to give this version final approval.

Are sufficient details of methods and materials provided to allow replication by others?

Partly

Is the rationale for creating the dataset(s) clearly described?

Yes

Are the datasets clearly presented in a useable and accessible format?

Partly

Are the protocols appropriate and is the work technically sound?

Yes

Reviewer Expertise:

Virology

I confirm that I have read this submission and believe that I have an appropriate level of expertise to confirm that it is of an acceptable scientific standard.

# Peer review: Reviewer response for version 1

**DOI:** 10.5256/f1000research.22537.r78179

**Published:** 2021-02-22

**Authors:** Marie-Eve Tremblay

**Affiliations:** 1 Division of Medical Sciences, University of Victoria, Victoria, British Columbia, Canada

This supplemental material provides additional transmission electron microscopy pictures in support of the presence of viral-like particles in the brain stem of post-mortem Parkinson's patient samples (compared with controls). 

 The rationale for providing additional pictures should be explained a little further, considering that these additional pictures do not provide more support to the original evidence published by the authors. In particular, it would have been useful to perform immunostaining or use additional validation methods to confirm that some of the viral-like particles are indeed viral-like particles and not something else (e.g. glycogen granules, which have a similar size and can also associate with the endoplasmic reticulum). 

 If presented as supplemental material, the pictures should have more annotations (e.g. arrows, pseudo-colours, etc.) to help readers visualize the structures of relevance. I would also recommend removing pictures showing glial processes, myelin, neurons (of note, sometimes neurons looked like astrocytes, it would be important to verify the ultrastructural identification criteria for the dataset), focusing on the topic of investigation: the viral-like particles.

 In addition, the ultrastructural preservation of the samples is generally suboptimal, which could be accounted for by the most-mortem interval or the pH of the cerebrospinal fluid, known to affect brain ultrastructure. More information regarding the samples could be useful to help interpret the dataset.

Are sufficient details of methods and materials provided to allow replication by others?

Yes

Is the rationale for creating the dataset(s) clearly described?

Partly

Are the datasets clearly presented in a useable and accessible format?

No

Are the protocols appropriate and is the work technically sound?

Partly

Reviewer Expertise:

brain ultrastructure, microglia, neurons, synapses, animal models, human post-mortem samples, stress, aging, neurodegenerative diseases

I confirm that I have read this submission and believe that I have an appropriate level of expertise to confirm that it is of an acceptable scientific standard, however I have significant reservations, as outlined above.

# Peer review: Reviewer response for version 1

**DOI:** 10.5256/f1000research.22537.r66787

**Published:** 2020-08-20

**Authors:** Doris Bucher

**Affiliations:** 1 Department of Microbiology and Immunology, New York Medical College, Valhalla, NY, USA

R.R. Dourmashkin and colleagues provide additional TEM images from their work on Parkinson’s Disease (PD). Earlier this group detected enterovirus antigen in PD brainstem tissue.

 Dourmashkin *et al. *show additional TEM of virus-like particles 30 nm diameter (VLPs) associated with endoplasmic reticulum membranes from PD cases. In nuclei of neurons from PD cases VLPs of 40-50 nm diameter were found. Similar particles were also found in polio cases.

 Lewy bodies were also found.

 Cytoplasmic ribosomes of 17 nm were observed in control neurons.

 It is valuable to the scientific community and PD researchers to have access to this supplemental material.

 Overall, need to improve labelling of figure legends by use of arrows pointing to key features.

**PD Brain:**

- Fig. 1-4
- Fig. 5 use arrow to i.d. VLPs assoc. with cytoplasmic inclusion body.
- Fig. 7., Fig. 8, Fig. 9—need arrows.
- Fig. 13—need arrow.
- Fig. 14 –NM not labelled.
- Fig. 16—VLP no arrows.
- Fig. 31, 32 –need arrows.
- Fig. 37--  same area as Fig. 36.
- Fig. 51—need arrows.
- Fig. 63—need arrows.
- Fig. 66, Fig. 67. Fig. 68—difficult to see cytoplasmic membrane—need arrows.
- Fig. 72, 73—need arrows.
- Fig. 77—need arrows.
- Fig. 79, 80, 81—need arrows.
- Fig. 84—need arrows.

 

**Poliomyelitis Spinal Cord:**—figures are out of order….

- Fig. 1, Fig. 2—need arrows.
- Fig. 6, 7, 8—need arrows.
- Fig. 11—need arrows.

 

**Control Brain (Ribosomes, etc.):**

- Fig. 1—need arrows.
- Fig. 7,8,9,10,11—need arrows.

Are sufficient details of methods and materials provided to allow replication by others?

Partly

Is the rationale for creating the dataset(s) clearly described?

Yes

Are the datasets clearly presented in a useable and accessible format?

Partly

Are the protocols appropriate and is the work technically sound?

Yes

Reviewer Expertise:

Virology

I confirm that I have read this submission and believe that I have an appropriate level of expertise to confirm that it is of an acceptable scientific standard, however I have significant reservations, as outlined above.
